# Complete Genome Sequence of *Actinobacillus pleuropneumoniae* Strain KL 16 (Serotype 1)

**DOI:** 10.1128/genomeA.01025-17

**Published:** 2017-09-21

**Authors:** Byung-Sun Park, Jemin Han, Dong-Ju Shin, Young-Ju Jeong, Nakhyung Lee

**Affiliations:** Technology Institute, KBNP, Inc., Dongan-gu, Gyeonggi, South Korea

## Abstract

*Actinobacillus pleuropneumoniae* is a bacterial pathogen causing highly contagious porcine pleuropneumonia. Due to limited information on this species, it is difficult to study the biology of *A. pleuropneumoniae* at the genome level. Here, we report the fully annotated genome sequence of *A. pleuropneumoniae* strain KL 16.

## GENOME ANNOUNCEMENT

*Actinobacillus pleuropneumoniae* is the etiological agent causing porcine pleuropneumonia, a highly contagious respiratory disease that results in considerable economic losses to the swine industry. To date, 15 serotypes have been reported worldwide, and their distributions vary geographically. Among them, only a few serotypes have been recognized to be highly pathogenic in swine. Such a difference is associated with some virulence factors, including lipopolysaccharide, outer membrane proteins, capsules, and Apx exotoxins (1). Serotype 1, a group of highly virulent pathogens, predominantly prevails and has caused major respiratory disease outbreaks on swine farms in South Korea and other countries. Interestingly, earlier studies reported that a strong protection against *A. pleuropneumoniae* was induced in the animals that were vaccinated with homologous serotypes but not with heterologous ones, indicating the importance of a serotype-specific protective immunity (2, 3). Because there is yet no vaccine against all serotypes, it has long been of interest to seek a highly effective vaccine that is capable of controlling multiple or all serotypes. For this goal, the whole-genome sequences of new isolates originating from different regions should be compiled for researchers to further investigate the genetic function of *A. pleuropneumoniae*.

Here, we present the complete genome of *A. pleuropneumoniae* strain KL 16, an isolate of serotype 1 prevalent in South Korea in 2016. It was grown in brain heart infusion medium supplemented with 0.01% NAD at 37°C. Serotyping was determined by PCR, and pathogenicity was confirmed by *in vitro* and *in vivo* experiments. The total DNA of the isolate was extracted and subjected to whole-genome sequencing using the PacBio RS II platform according to the supplier’s protocol. The primary sequencing yielded a total of 1,390,886,435 bp with 156,851 reads. After filtering short reads, the obtained reads were assembled with the Hierarchical Genome Assembly Process (HGAP) and polished with Quiver. The final assembled genome contained two circular contigs, including a chromosome and a plasmid. The assembled genome was automatically annotated with the Prokka software program. The chromosome comprises 2,357,806 bp with a GC content of 41.2%, which is similar to that of other reported genomes (1, 4–6); the plasmid comprises 7,699 bp with a GC content of 60.9%. The chromosome included 2,199 putative protein-coding sequences (CDSs), 62 tRNAs, and 19 rRNA-related genes. However, the single plasmid revealed only 10 putative CDSs without antibiotic resistance genes. According to earlier studies, it has been reported that the pathogenicity and immunity of *A. pleuropneumoniae* were considerably variable among serotypes, even among the same serotypes of different geographic origin (2, 3). It is of interest to investigate such differences in order to explore potential protective antigens for developing a universal vaccine against all serotypes. Thus, the availability of the genome sequence of new isolates will provide insight for further genome-based studies, such as those related to epidemiology, diagnostic tools, and the development of vaccines.

### Accession number(s).

The whole-genome sequence of *A. pleuropneumoniae* KL 16 has been deposited at GenBank under accession numbers CP022715 (chromosome) and CP022716 (plasmid).
